# HaloDom: a new database of halophiles across all life domains

**DOI:** 10.1186/s40709-017-0072-0

**Published:** 2018-01-15

**Authors:** Alexios Loukas, Ilias Kappas, Theodore J. Abatzopoulos

**Affiliations:** Department of Genetics, Development & Molecular Biology, School of Biology, Aristotle University of Thessaloniki, 54124 Thessaloniki, Greece

**Keywords:** Online database, Salinity, Extremophiles, Extreme environments, Tolerance

## Abstract

**Background:**

Halophilic organisms may thrive in or tolerate high salt concentrations. They have been studied for decades and a considerable number of papers reporting new halophilic species are being published every year. However, an extensive collection of these salt-loving organisms does not exist nowadays. Halophilic life forms have representatives from all three life domains, Archaea, Bacteria and Eukarya. The purpose of this study was to search for all documented halophilic species in the scientific literature and accommodate this information in the form of an online database.

**Results:**

We recorded more than 1000 halophilic species from the scientific literature. From these, 21.9% belong to Archaea, 50.1% to Bacteria and 27.9% to Eukaryotes. Our records contain basic information such as the salinity that a particular organism was found, its taxonomy and genomic information via NCBI and other links. The online database named “HaloDom” can be accessed at http://www.halodom.bio.auth.gr.

**Conclusions:**

Over the last few years, data on halophiles are growing fast. Compared to previous efforts, this new halophiles database expands its coverage to all life domains and offers a valuable reference system for studies in biotechnology, early life evolution and comparative genomics.

## Background

Halophiles are extremophile or extremotolerant organisms that can survive in high salinity. They are categorized as slight, moderate and extreme, depending on their maximum salinity tolerance [1]. Halophilic species exist across all life domains [2, 3] showing considerable diversity in metabolic strategies and physiological responses, especially among microbes [1, 4–6]. Research on halophiles has mainly focused on the specific adaptations and molecular mechanisms that enable them to maintain their osmotic balance under salt-stress [7–9]. A great deal of interest has also been channeled towards the investigation of their diversity and phylogenetic relationships as the highest majority of them constitute ancient evolutionary lineages [10, 11]. On a different avenue, biotechnology has recently decided to delve deep into the survival kits of extremophiles in the hunt of biocatalysts functioning in hostile environments. All this interest is reflected in the plethora of papers reporting new halophilic species every year [12–14], a trend which is expected to increase. As a consequence and due to the large quantities of data produced by next-generation sequencing, there is a need for a database repository of extremophiles which will be regularly updated.

So far, there are three halophilic databases available online: HaloWeb [15], HaloBase [16] and HProtDB [17]. HaloWeb focuses on genome information and provides complete genome sequences available for downloading. There are also features like blasting sequences against a genome and genomic maps. There are 10 haloarchaeal species registered in total. HaloBase contains more general information in 23 halophilic archaeal and bacterial halophiles. GenBank sequence numbers, number of chromosomes and plasmids, gene/protein content and cellular features are among the database entries. HaloBase provides user accounts, followed by the ability to add a new organism as a registered member. In HProtDB, the first priority is protein content. The resource contains physical and biochemical properties of halophilic proteins for 21 strains of Archaea and Bacteria. It also allows users to register as members and enter their own halophilic data. All three databases are restricted to information about halophilic Archaea and Bacteria, their number of entries is limited to an average of 18, and are irregularly updated.

In this work, we report on a new halophiles database covering more than 1000 halophilic species and spanning all life domains. This new resource named “HaloDom” can be accessed at http://halodom.bio.auth.gr.

## Methods

An extensive literature search has been carried out through the Web of Science, Scopus, PubMed and Google Scholar using appropriate keywords (i.e. haloph*, salt, saline, hypersaline, extremophile) as well as combinations of them. Ultimately, the Web of Science was chosen as the primary source of literature as it provided a sophisticated search/query engine that suited our methodology and was proven to contain most of the papers found in other literature databases. The keyword combination that returned most papers in Web of Science was “sp nov haloph*” (on title section), returning 610 papers reporting new halophilic species up to 2017. The same keyword combination returned many results in Google Scholar (2410), but not all of these papers contained the desirable keywords in their titles making its search engine unsuitable for our purposes. Scopus returned 615 results, but the interface of Web of Science offered a more flexible environment. There was great overlap among all three databases. Google Scholar however also returned a lot of unrelated papers. Finally, a small number of books and reports containing useful information about halophilic species (albeit with no salinity data) were also included.

The obtained results were initially refined by topic and document type and further filtered out manually. The final dataset was retrieved as a tab-delimited format file and then loaded to a spreadsheet organized in several columns (i.e. full taxonomy of each species, salinity record or range, halotolerance classification, genome availability, bibliography, notes/other information). Several taxonomy databases were used for registering the taxonomy of halophilic organisms (Table 1). “Salinity recorded or range” column reports a single salinity value, a range of salinities or both depending on the available information from the scientific source. “Halotolerance classification” included three halophilic categories: *slight*, *moderate* and *extreme*. We searched for full genomes for all our entries in NCBI genome database. The column “Genome availability” contained five possible states: *complete genome*, *shotgun*, *mitochondrial genome*, *chloroplast genome* and *no* (not available). “Bibliography” contained the scientific article/s from which the halophilic information was extracted. “Notes/other info” is a complementary column for any type of information or metadata gauged as necessary to be documented.

**Table 1:** Taxonomy databases that were used to record taxonomic information about halophiles in HaloDom

Taxonomy database	Number of species
NCBI taxonomy browser	942
algaeBASE	49
World Register of Marine Species	32
Encyclopedia of Life	32
Integraded Taxonomic Information System	17
Atlas of Living Australia	11
Catalogue of Life	1
Global Biodiversity Information Facility	1
Global species	1
INPN—Inventaire National du Patrimoine Naturel	1
Marine species identification portal	1
Sum	1088

The spreadsheet was converted to a comma separated values file (csv) and uploaded to a local database with the use of XAMPP and apache server [18]. PhpMyAdmin was also used [19] which is a tool intended to handle the administration of the MySQL database protocol locally or in a webpage. Additionally, NetBeans 8.1 IDE (Integrated Development Environment) [20] was installed for creating the website with the use of HTML (HyperText Markup Language) and the programming languages PHP and Javascript. The user interface was created and modified using HTML and cascading style sheets (CSS) for the visual parts, and both PHP and Javascript for all functional parts regarding interactions between users and the database.

After importing the spreadsheet to the database all data were converted from a csv file to a table called “halodb”. The table was assigned with a primary key column called “Species_ID”. A primary key in mySQL is a number for each individual row of a table and it is unique. In this case, every halophilic species has a unique primary key. This primary key, or “Species_ID” column, always contains an integer starting from 1 and set to “auto-increment”. As more species are added to the database, this number is automatically increased providing every species with its distinctive ID number.

HaloDom’s data structure started as one table that contained all information. However, as data volume increases it is necessary to break down the database into several tables. This methodology improves the speed and efficiency of the database during user query. It is also a way of organizing data, so that administrators can easily check the data integrity, make changes and reduce redundancy. The structure of the database was changed from the table called “halodb”, containing all recorded information, to three tables. The first information separated from “halodb” was the “Bibliography” column, which moved to a table called “Bibliography”. “Bibliography” table was assigned a primary key called “Biblio_id” and four columns: “pub_title” which contains the title of the study, “authors” containing the study’s author/s, “journal” mentioning the name of the journal and “biblio_link” providing a direct link to the study.

**Fig. 1:**
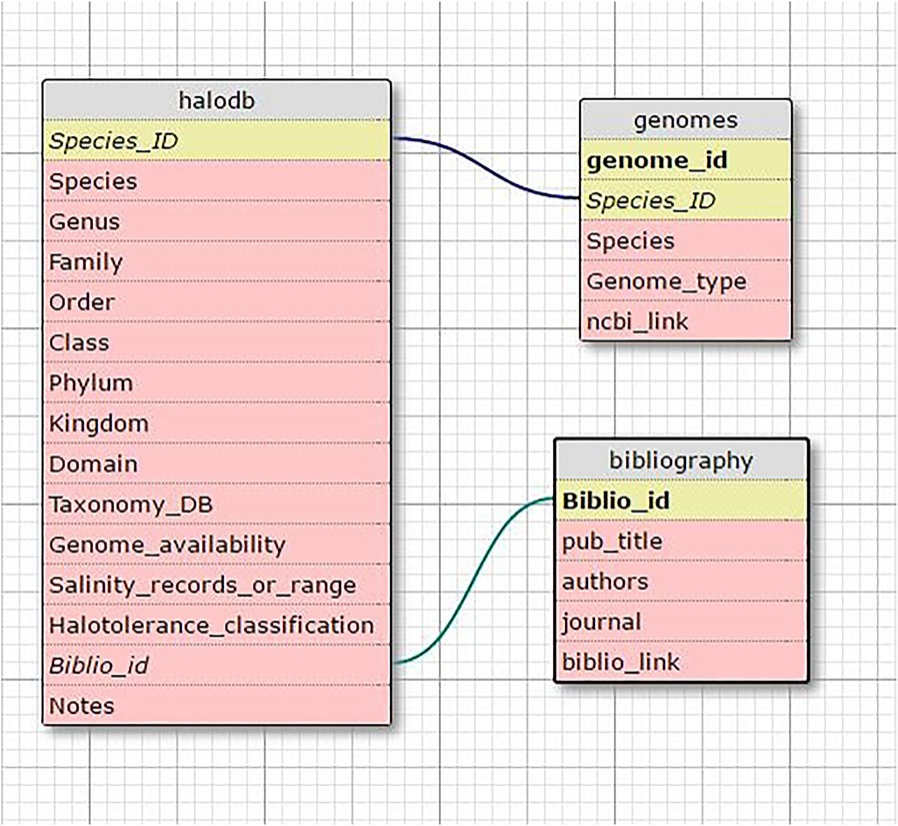
HaloDom consists of three tables. Foreign key relationships are shown with blue and green lines. Yellow cells are integer numbers while pink cells are varchar, meaning mixed characters and numbers

The third table is called “genomes” and contains five columns. “Genome_id” which is the primary key, “Species_ID” which is a foreign key from “halodb” table, “Species” which is the species name, “Genome_type” which declares the type of genome and the “ncbi_link” which contains the link to the genome details in the NCBI genome database. A graph of the relationships between all three tables can be found in Fig. 1.

The website project in NetBeans 8.1, written mostly on HTML, CSS and PHP, was named “HaloDB”. Several .php files were created in order to design the user interface and database functions. The home page contains a welcoming text and a photo slide created with the use of a jQuery script. Moreover, pie charts were created with Google Charts [21] and the use of JavaScript. These charts were embedded to the webpage code and can be viewed through the user interface. Halophilic entries are presented in a new page when clicked, where all available information is listed. Additionally, if a full genome is available the user can be redirected to the corresponding NCBI genome page. Also users can perform a nucleotide or protein search.

## Results

**Fig. 2:**
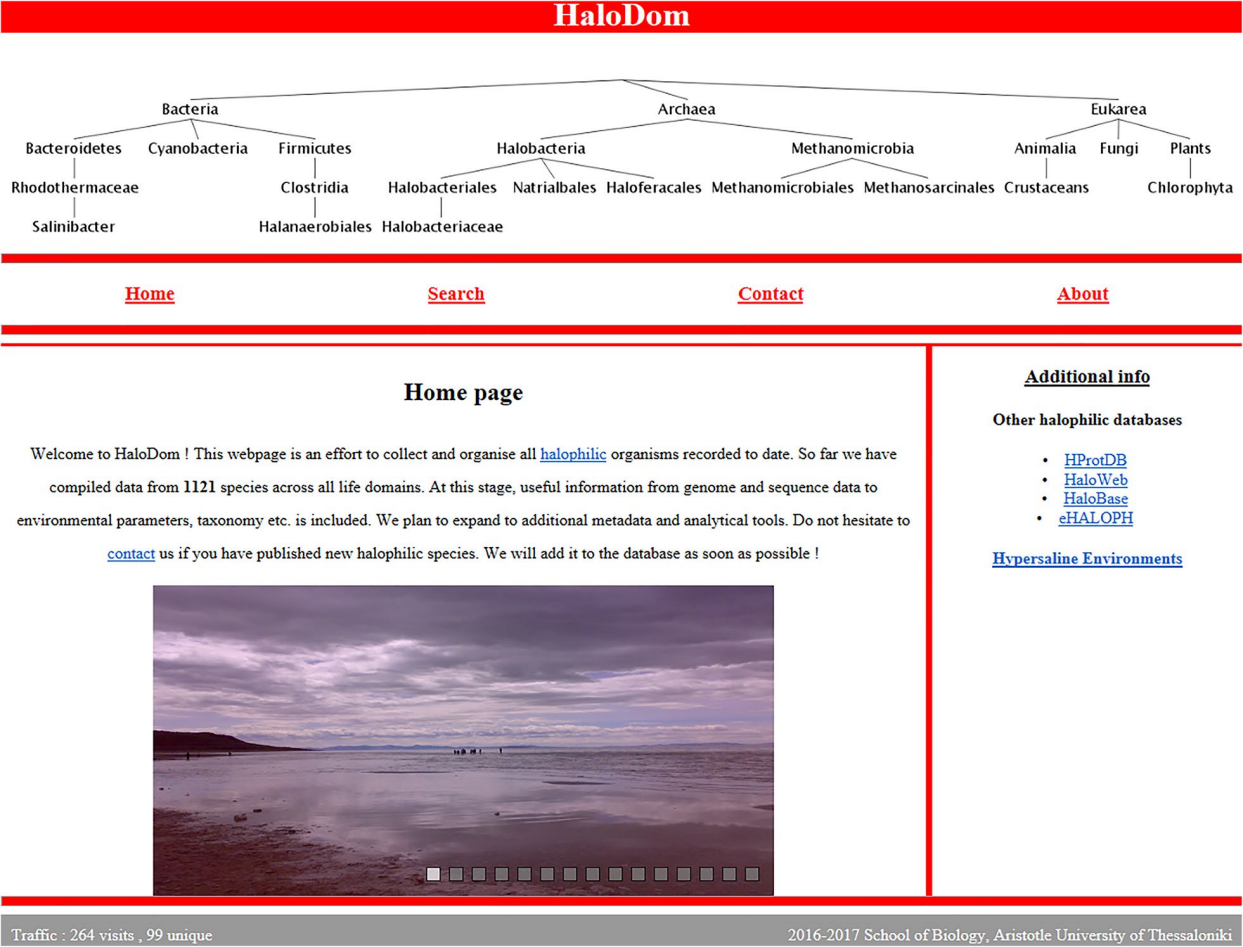
Homepage of HaloDom contains a welcoming text, a small tree graph and a photo slide

We designed HaloDom, an online database containing more than 1000 halophilic species from all life domains. Users are able to perform a keyword search in all columns of the “halodb” table and retrieve all matching entries in numbered order. The homepage of HaloDom can be seen in Fig. 2.

**Fig. 3:**
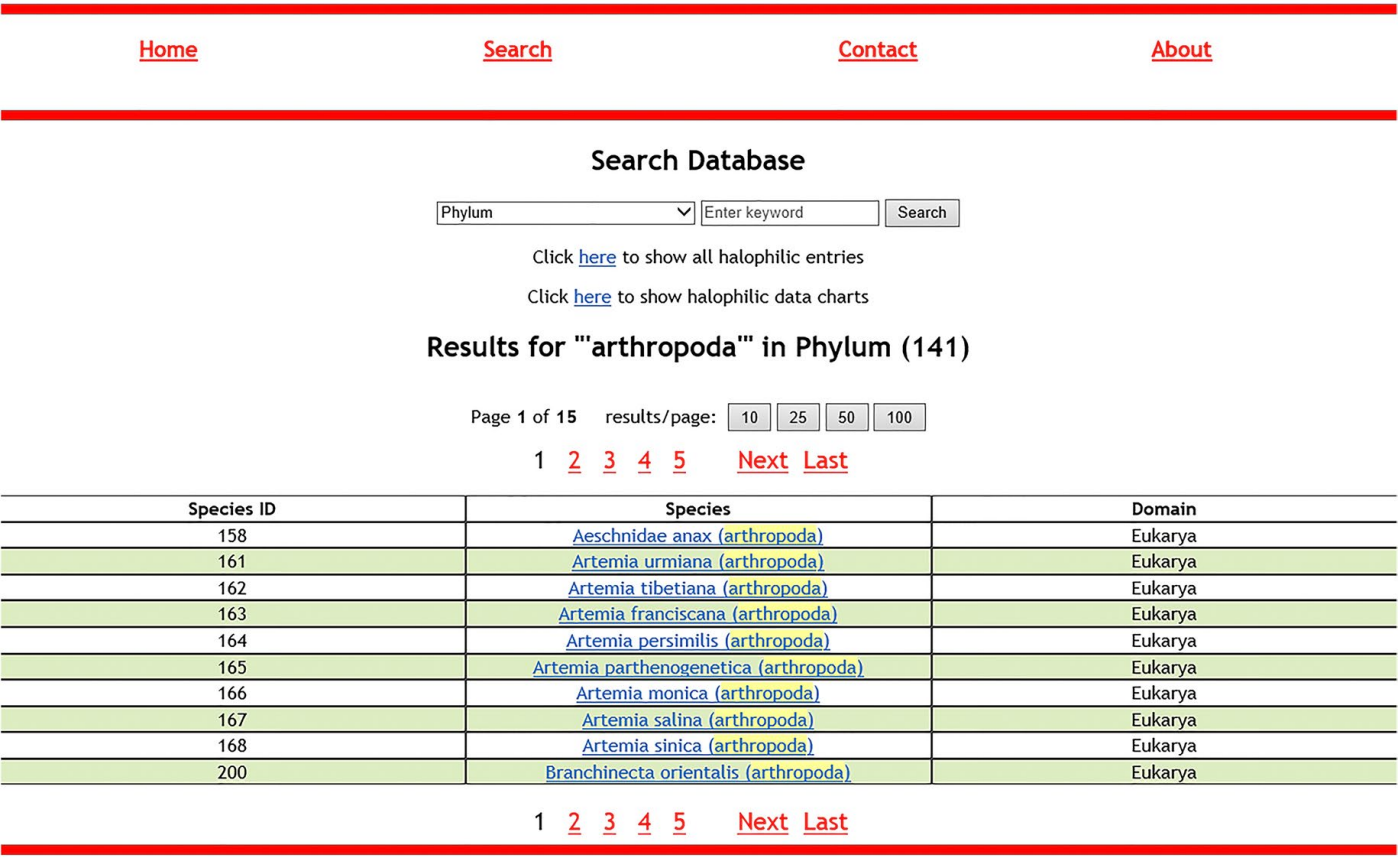
The first 10 results for keyword “arthropoda” in the “Phylum” column

**Fig. 4:**
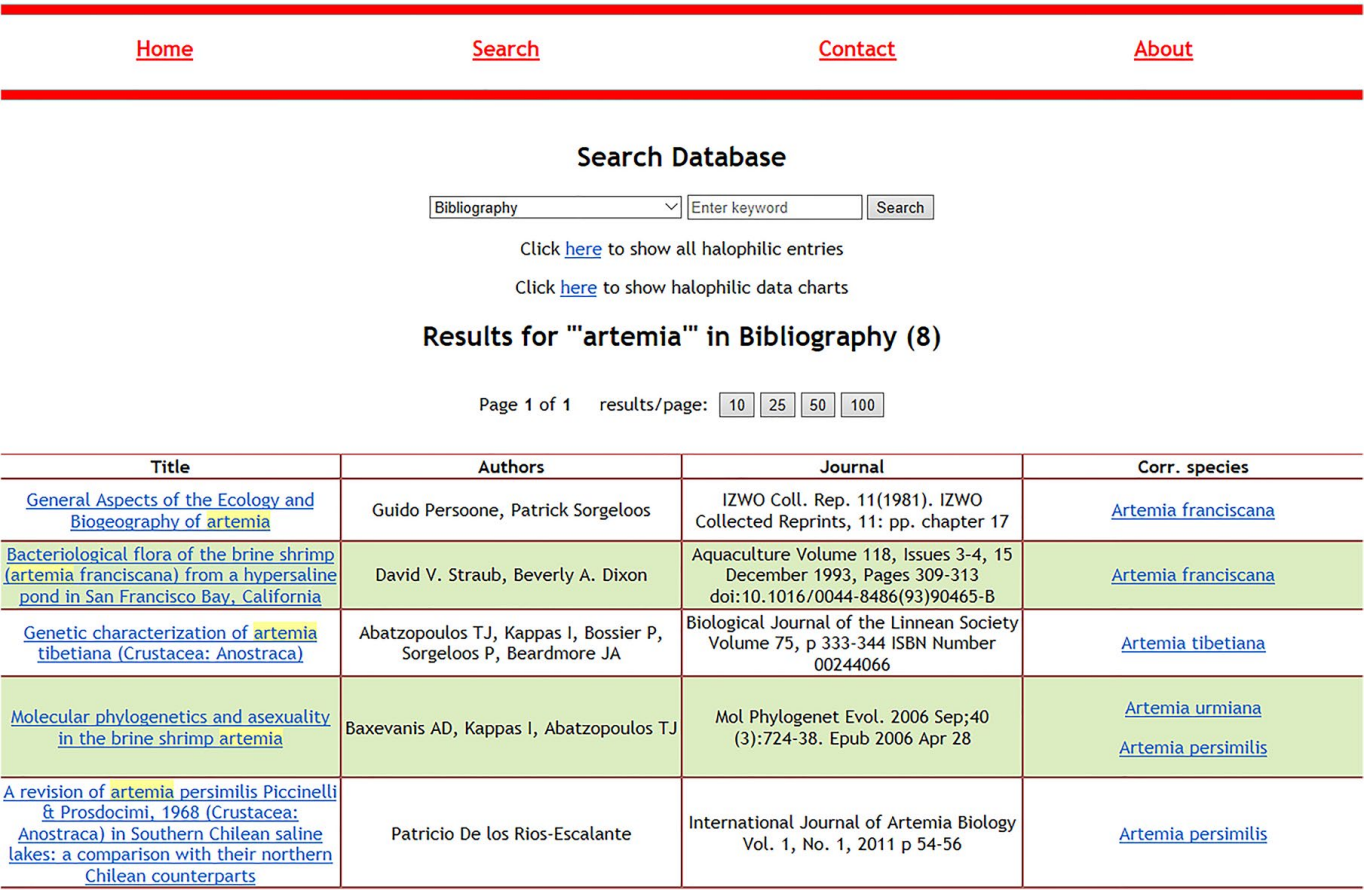
The displaying format of “Bibliography” field results for the keyword “artemia”

**Fig. 5:**
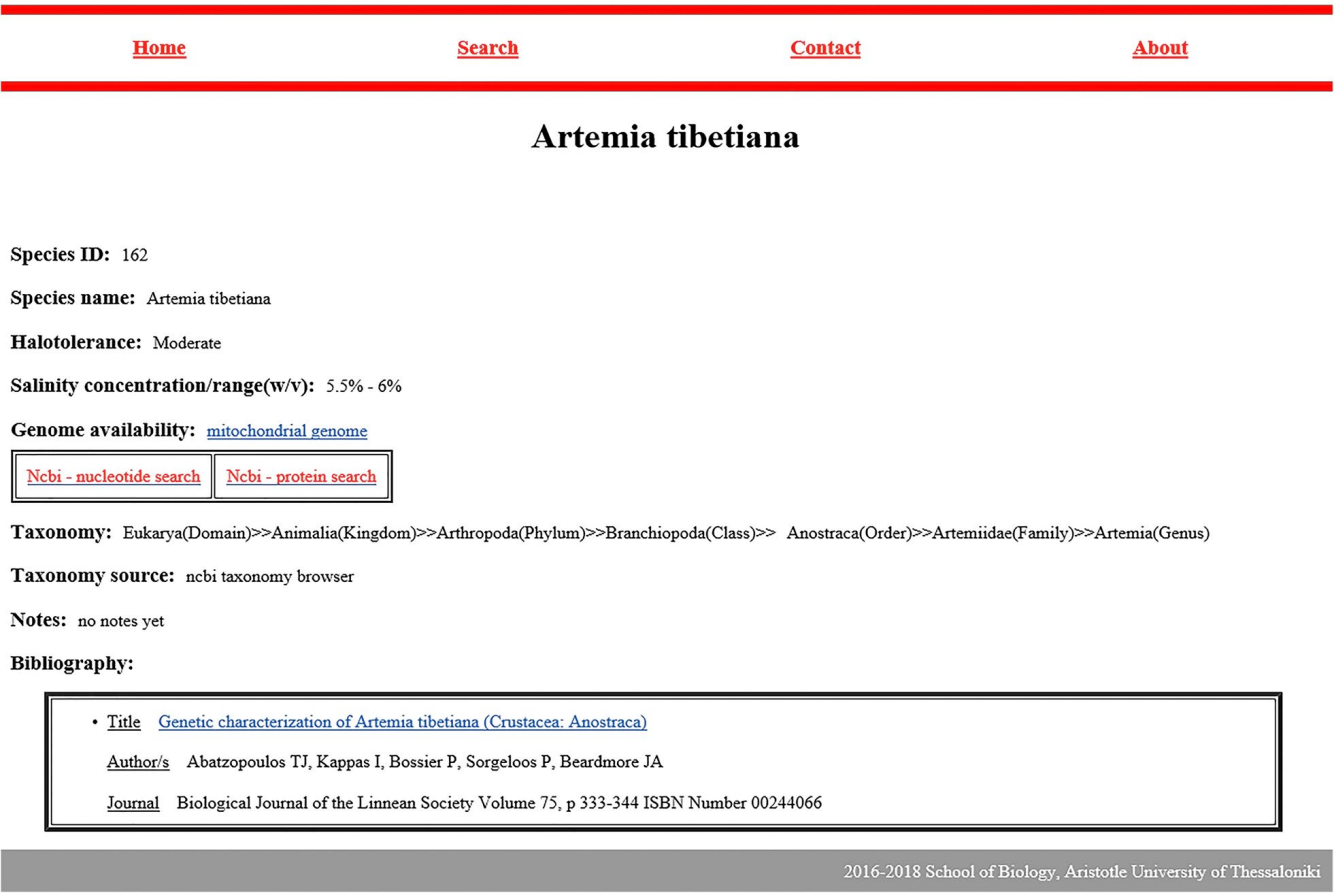
The entry page for *Artemia tibetiana*

The main menu contains four options: “Home”, “Search”, “Contact” and “About”. The search page, apart from retrieving data entries, can also show all recorded data, and several pie charts created for a better visual interpretation of the listed halophilic data. The search page prompts the user to choose a column and perform a keyword search. When displaying the results, search always displays “Species_ID”, “Species” and “Domain” columns. The column that the user selected to perform the keyword search is shown in parentheses inside the “species” column. Exact or partial keyword matches are highlighted as light-colored text. The results are displayed in several pages, if necessary. Users can choose how many results per page should be displayed (10, 25, 50, 100). When a search is performed on “Bibliography” field, the results are shown on a different table that contains paper title, authors, journal and corresponding species. Figure 3 shows the search results page for all fields except “Bibliography” while Fig. 4 shows the results table for “Bibliography” searches. The species name is always clickable and leads to the corresponding entry. The entry page contains all available information and can lead the user to NCBI for more genomic information. Figure 5 displays an example entry page for *Artemia tibetiana*.

**Fig. 6:**
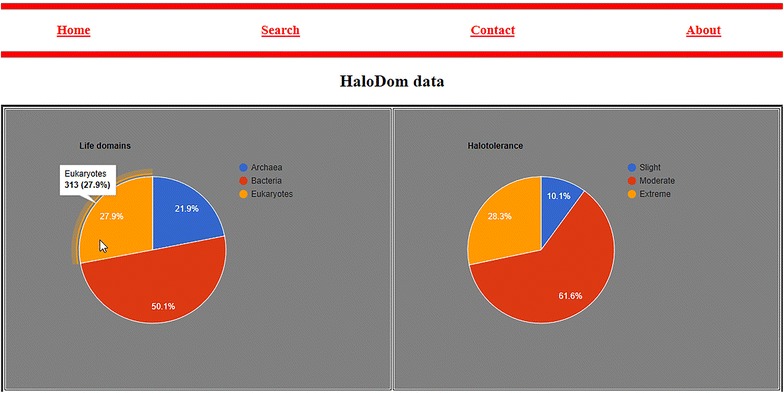
Halophilic data pie charts. On the left side: frequency and percentage of Archaea, Bacteria and Eukaryotes. On the right side: frequency and percentage of slight, moderate and extreme halophiles in the database

When showing all data from the search page, the user is able to select ascending or descending order with respect to a certain column. The pie charts visualize basic information about the data. For example, the first chart calculates the percentage of Archaea, Bacteria and Eukarya in our database. When the user’s mouse hovers above a certain piece, the frequency is shown first and then the corresponding percentage enclosed in parentheses. The first two pie charts are shown in Fig. 6.

“Contact” section lists the administrators and contact information. “About” page shows the date of creation of HaloDom, current number of registered halophilic species and the database version.

## Discussion

We present HaloDom, a database hosting information on more than 1000 halophilic species. This new resource expands considerably compared with previous databases in terms of coverage (representatives from all life domains) and number of entries. Periodical updates are scheduled once every 2 months and as the database grows additional metadata (e.g. geographic distribution, biochemical properties etc.) and analytical tools are planned to be incorporated. For database expansion, we envisage summoning an international panel of experts on extremophiles and engaging the international community from various fields.

Occasionally, during data curation and annotation, species nomenclature proved to be a challenge. This was especially true for Archaea and Bacteria given their notoriously difficult taxonomy and the fast discovery of new strains [22]. We invested considerable efforts into resolving this issue by using several taxonomy databases (see Table 1) but we also encourage user feedback. A grey picture also exists in the literature regarding threshold values in halophile classification (slight/moderate/extreme). For example, in one study the copepod *Cletocamptus retrogressus* was found in 2–7.4% (w/v) salinity, and thus categorized as slight to moderate halophile, while in another study the recorded salinity range was 19.8–36% (w/v), characteristic of extreme halophiles. This probably reflects the limited knowledge on the biology of many species but as additional data are gathered more accurate annotations are expected. Also, in the light of idiosyncratic molecular mechanisms and signatures in extreme halophilic Archaea [7, 23], criteria for halophile classification could be refined.

The current database can be used as a useful repository and starting point for a wide range of research topics. Over the last few years, investigations have focused on the mechanisms responsible for modulating survival in hypersaline settings [3, 24–28], on the biotechnological production of halophile macromolecules [29, 30], on the phylogenetic position of halophiles in the tree of life [31], on climate change [32, 33] and even on astrobiology [34]. It is therefore obvious that halophile research addresses appealing questions to several fields of biology, especially in combination with the diverse spectrum of extremophile organisms. As pointed out by de Lorenzo [35], extremophiles reframe the window of viability. The answer to the basic question whether sustaining life in physicochemical extremes is a matter of entire adaptation or due to the action of a few genes is crucial, multidisciplinary and influential.
